# Effect of Amelogenin Coating of a Nano-Modified Titanium Surface on Bioactivity

**DOI:** 10.3390/ijms19051274

**Published:** 2018-04-24

**Authors:** Chisato Terada, Satoshi Komasa, Tetsuji Kusumoto, Takayoshi Kawazoe, Joji Okazaki

**Affiliations:** 1 Department of Removable Prosthodontics and Occlusion, Osaka Dental University, 8-1, Kuzuhahanazono-cho, Hirakata-shi, Osaka 573-1121, Japan; terada-c@cc.osaka-dent.ac.jp (C.T.); joji@cc.osaka-dent.ac.jp (J.O.); 2 Osaka Dental University Japan Faculty of Health Sciences, 1-4-4, Makino-honmachi, Hirakata-shi, Osaka 573-1144, Japan; kusumoto@cc.osaka-dent.ac.jp; 3 Osaka Dental University, 8-1, Kuzuhahanazono-cho, Hirakata-shi, Osaka 573-1121, Japan; kawazoe@cc.osaka-dent.ac.jp

**Keywords:** amelogenin, nanostructure, host-implant interaction, tissue regeneration

## Abstract

The interactions between implants and host tissues depend on several factors. In particular, a growing body of evidence has demonstrated that the surface texture of an implant influences the response of the surrounding cells. The purpose of this study is to develop new implant materials aiming at the regeneration of periodontal tissues as well as hard tissues by coating nano-modified titanium with amelogenin, which is one of the main proteins contained in Emdogain^®^. We confirmed by quartz crystal microbalance evaluation that amelogenin is easy to adsorb onto the nano-modified titanium surface as a coating. Scanning electron microscopy, scanning probe microscopy, X-ray photoelectron spectroscopy, and Fourier-transform infrared spectroscopy analyses confirmed that amelogenin coated the nano-modified titanium surface following alkali-treatment. In vitro evaluation using rat bone marrow and periodontal ligament cells revealed that the initial adhesion of both cell types and the induction of hard tissue differentiation such as cementum were improved by amelogenin coating. Additionally, the formation of new bone in implanted surrounding tissues was observed in in vivo evaluation using rat femurs. Together, these results suggest that this material may serve as a new implant material with the potential to play a major role in the advancement of clinical dentistry.

## 1. Introduction

Titanium dental implants have been widely used as biomaterials to replace missing teeth, with ossointegration currently considered as the optimal implant-to-bone interface [1]. Natural teeth are attached to the surrounding alveolar bone via soft periodontal ligament tissues, which are critical for the function and eruption of native teeth. In comparison, conventional dental implants are anchored to alveolar bone through osseointegration without the formation of cementum and periodontal regiment cells. As osseointegrated implants are “ankylosed” and do not exhibit the same mobility as natural teeth having a periodontal ligament, efforts have been made during the past few decades to compensate for this obvious difference by integrating “shock absorbing systems” into the implant or its superstructure [2,3]. Thus, regeneration of the cementum and periodontal ligament tissue on the surface of titanium implants would afford significant clinical implication. However, although some studies have attempted to propagate periodontal ligament cells on the surface of titanium and then implanted the titanium abutment in vivo [4,5,6], such strategies are not practical because the required primary cell culture is costly and immunorejection associated risks.

An alternative approach is to facilitate the early bone differentiation of mesenchymal cells and improve osseointegration on titanium surfaces [7,8,9,10,11]. The surface characteristics of an implant material can affect its own rate and extent of osseointegration; in large part because material surface properties and structures play important roles in the adsorption of proteins, which might influence cell behavior. Accordingly, studies have demonstrated that certain surface-modified materials are also highly effective for the adhesion, growth, and osteogenic differentiation of cells [12,13,14]. In particular, nanostructural modification can accelerate hard-tissue engineering through increased initial cell attachment to the surface [15]. Surface modification may thus allow the osseointegration period of any implant material to be shortened. Specifically, surface roughness has been shown to influence the initial cellular response; for example, the differentiation of osteoblastic cells through α5 integrin interactions [16]. 

It has been demonstrated that TiO_2_ nanotubes and titania nanosheets (TNS), which are similar nanostructures generated via titanium deposition by TiO_2_ sputtering [17], can be formed on titanium surfaces by treatment in 10 M NaOH aqueous solution at 30 °C [17]. In particular, our laboratory showed that treatment with NaOH aqueous solution could produce a rough, nanoscale surface [18]. Previous studies reported that TNS produced by chemical processing promoted the osteogenic differentiation of rat bone marrow (RBM) cells [18,19,20,21]. Additionally, our research group demonstrated that TNS-modified titanium surfaces could promote bone differentiation of periodontal ligament cells, rat aortic endothelial cells (RAECs), to upregulate the expression of angiogenic factors and adhesion molecule genes, which play an essential role in controlling inflammation and revascularization during wound healing after implantation [22,23]. 

In addition, amelogenin, the predominant protein component of secretory stage tooth enamel, has been used to fabricate biomimetic coatings on titanium and bioglass surfaces [24,25,26]. Amelogenin constitutes a matrix protein secreted by ameloblasts that comprises 95% of the mineralizable matrix in the developing enamel. In turn, an enamel matrix derivative (EMD) is a commercial product used for regeneration of periodontal bony defects in patients with periodontal disease. To date, EMD represents the only agent that can restore periodontal defects by regenerating cementum, periodontal ligament, and alveolar bone [27,28,29]. Notably, recent studies have shown that purified amelogenin could affect regeneration of the tooth-supporting tissues around implant material [30,31]. We, therefore, hypothesized that an amelogenin coating on a titanium surface would promote osteoblastic differentiation and extracellular matrix production. However, the binding strength between titanium and amelogenin remained a concern. Thus, we further considered that a nano-modified titanium surface exhibiting a strong binding ability with protein could be used as an experimental material. Specifically, as it was inferred that the effects on the behavior of various cells manifested by TNS [22,23] derived from changes in the mechanical and chemical structure of the titanium surface, we considered that adsorption of proteins such as amelogenin would be very high on the TNS-modified titanium surface. 

The combination of amelogenin and TNS structure on titanium surface might, thus, be expected to facilitate the formation of hard tissues as well as to enhance periodontal tissue regeneration. The aims of the present study were therefore to investigate the effect of amelogenin coating applied on a TNS-modified titanium surface on the behavior of RBM cells and rat periodontal ligament (RPL) cells at both the in vitro and in vivo level. We expect that such further development of advanced implant materials using nanotechnology will be useful to improve their osseointegration and periodontal regeneration capabilities.

## 2. Results

### 2.1. Sample Preparation

**Figure 1:**
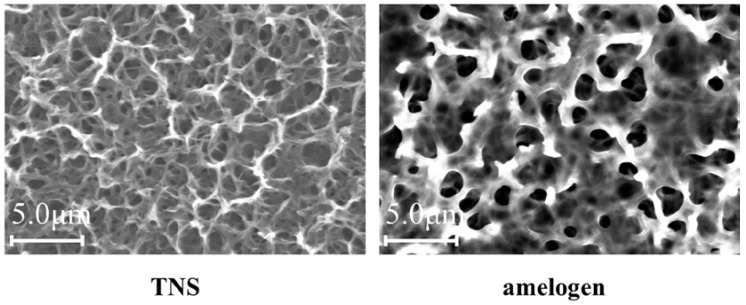
SEM micrographs of control and test groups.

**Figure 2:**
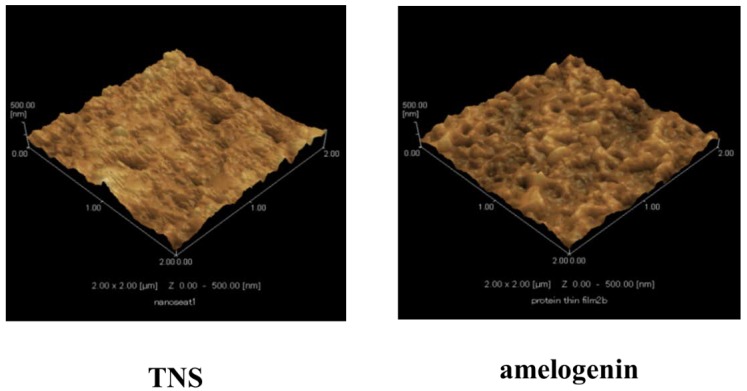
Scanning probe (SP) micrographs of control and test groups.

**Figure 3:**
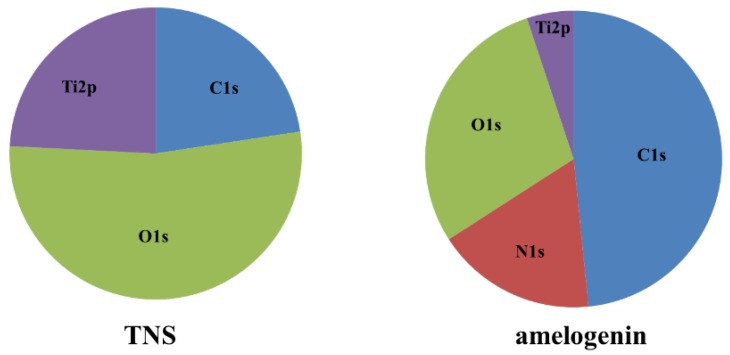
Wide scan of the test and control groups.

**Figure 4:**
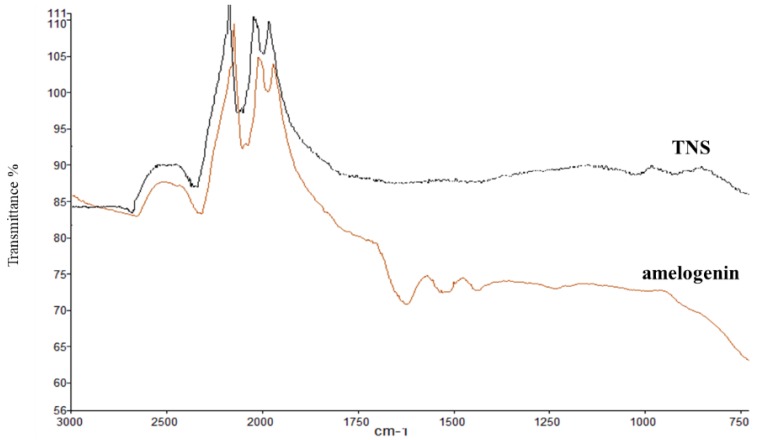
FTIR analysis of test and control groups.

Scanning electron microscope (SEM) analysis indicated that the TNS surfaces exhibited a nanoscale network structure after modification in NaOH at 30 °C in the control group (titanium disks and screw). In comparison, amelogenin coating was confirmed on the TNS surface in the test group (Figure 1). The surface morphologies of the test and control titanium surfaces are shown in Figure 2. The surface roughness (Ra) values were 42.495 and 13.775 nm for the titanium surfaces of the test and control groups, respectively. Figure 3 shows the results of wide-scan X-ray photoelectron spectroscopy (XPS) surface chemical analyses of test and control titanium disks. The presence of Ti, O, C, and Na was confirmed on the surfaces of test and control titanium disks. In addition, the presence of N was confirmed on the surface of the test group. The Fourier Transform Infrared Spectroscopy (FTIR) spectra of the test and control group are shown in Figure 4. In the test group, the FTIR spectrum of the sensor surface showed bands around 1500 cm^−1^ and 1650 cm^−1^ compared with the control group, which was similar to the FTIR spectrum (1650 cm^−1^ and 1450 cm^−1^) demonstrated by Warren et al. of bovine pancreatic trypsin inhibitor, representing the Amide I and Amide II bands. Thus, from the results of all the analyses, it was inferred that the material surface of the experimental group was coated with amelogenin.

### 2.2. Quartz Crystal Microbalance (QCM) Measurements

**Figure 5:**
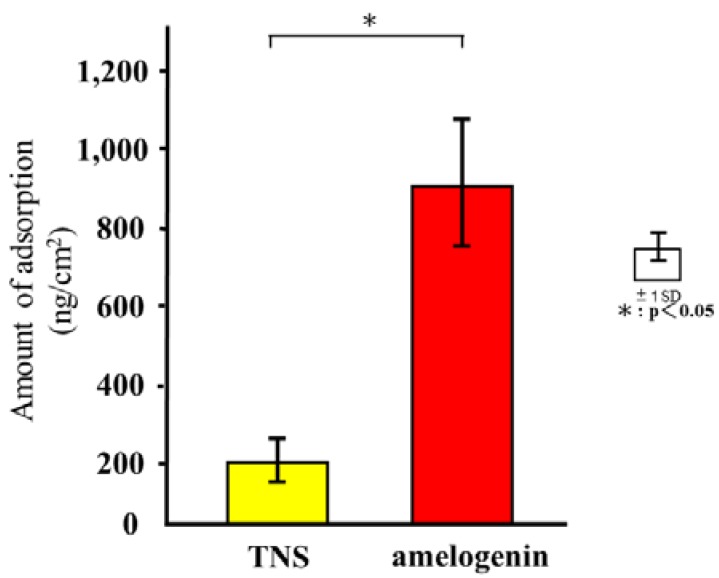
Quartz crystal microbalance (QCM) analysis of test and control groups. * *p* < 0.05.

Figure 5 shows the adsorption of amelogenin based on the test and control groups. An immediate decrease in frequency was observed after the injection of amelogenin, which was identified as indicative of the adsorption of amelogenin. The adsorption of albumin and amelogenin on the TNS-modified titanium QCM sensor produced a decrease in frequency that was greater than that measured for the Ti sensor. 

### 2.3. Cell Adhesion

**Figure 6:**
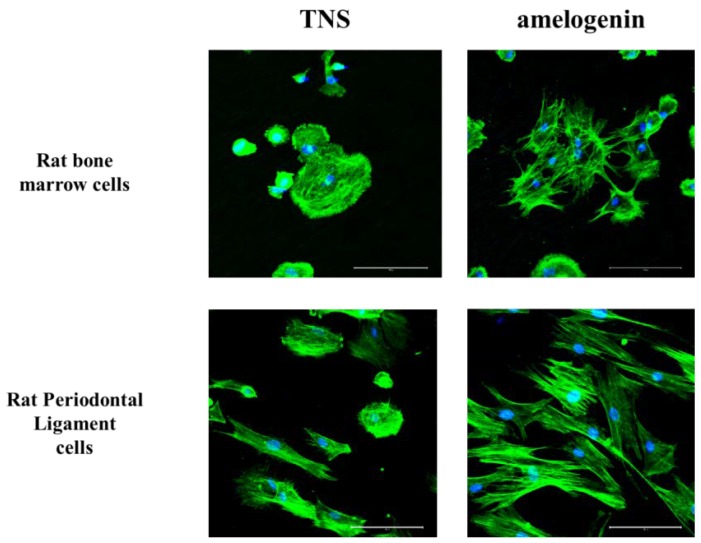
Morphology of rat bone marrow (RBM) cells and rat periodontal ligament cells in the control and test groups after culturing for 24 h. Actin filaments (green) were labeled with Alexa Fluor 488-phalloidin and nuclei (blue) were stained with 4′,6-diamidino-2-phenylindole (DAPI).

**Figure 7:**
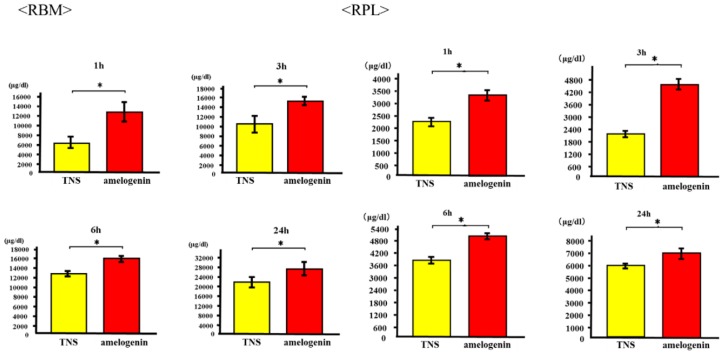
Cell adhesion in control and test groups. * *p* < 0.05.

RBM and RPL cell morphology was evaluated by Alexa Fluor 488-phalloidin and DAPI staining after 24 h of incubation. Compared to RBM and RPL cells attached to the TNS-modified titanium surface, those on the amelogenin-coated TNS-modified titanium surface showed greater F-actin expression and more filopodia and lamellipodia (Figure 6). RBM and RPL cell adhesion on the disks after 1, 3, 6, and 24 h of incubation was assessed (Figure 7). Significant differences were observed in cell adhesion and proliferation between the test and control samples for all time periods.

### 2.4. Amelogenin Induced RBM and RPL Cell Differentiation and Matrix Deposition on TNS-Modified Titanium Surfaces In Vitro

**Figure 8:**
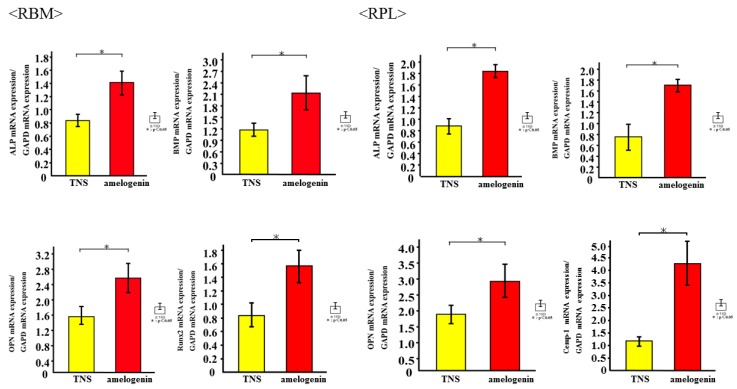
Quantitative real-time (qRT)-PCR analysis of osteogenesis and cementum related gene expression in control and test groups. * *p* < 0.05.

**Figure 9:**
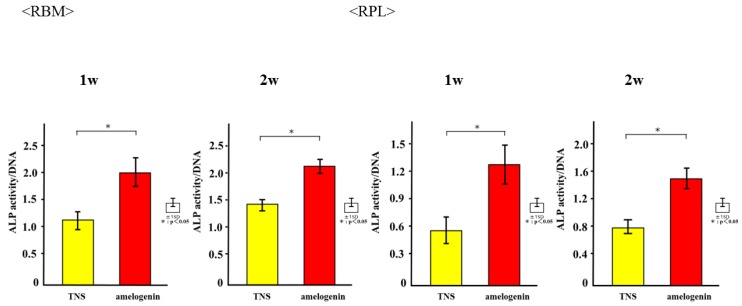
ALP activity in control and test groups. * *p* < 0.05.

**Figure 10:**
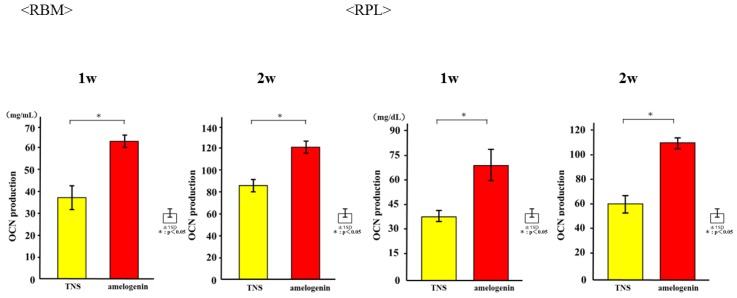
OCN production in control and test groups. * *p* < 0.05.

**Figure 11:**
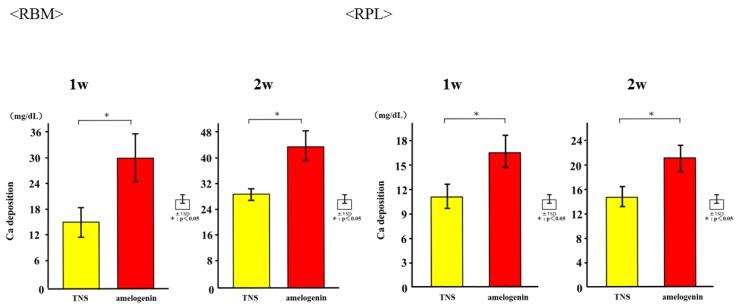
Ca deposition in test and control groups. * *p* < 0.05.

The mRNA expression levels of osteogenesis-related genes including Alp, Runx2, Bmp, and Opn in RBM cells and Alp, Bmp, Opn, and CEMP-1 mRNA in RPL cells grown on the different surfaces for 3, 7, 14, and 21 days were assessed by qRT-PCR. The levels of all genes were upregulated in cells grown on the test group titanium surfaces as compared to those grown on the control titanium surface (Figure 8). ALP activity of RBM and RPL cells grown on the different substrates for 7 days was found to increase with time, with significant differences being observed in ALP activity between the test and control titanium surfaces at 7 and 14 days (Figure 9). Measurement of OCN levels in the culture supernatants of RBM and RPL cells grown on the different substrates for 21 and 28 days demonstrated higher levels of OCN in the supernatant of cells grown on the test than on the control surfaces at both time points (Figure 10). Calcium deposition in cells cultured on the different surfaces for 21 and 28 days was also assessed as a measure of osteogenic differentiation. Mineralization was greater in cells grown on the test group than in those grown on the control group (Figure 11).

### 2.5. Amelogenin Induced Bone Differentiation on TNS-Modified Titanium Surfaces In Vivo 

**Figure 12:**
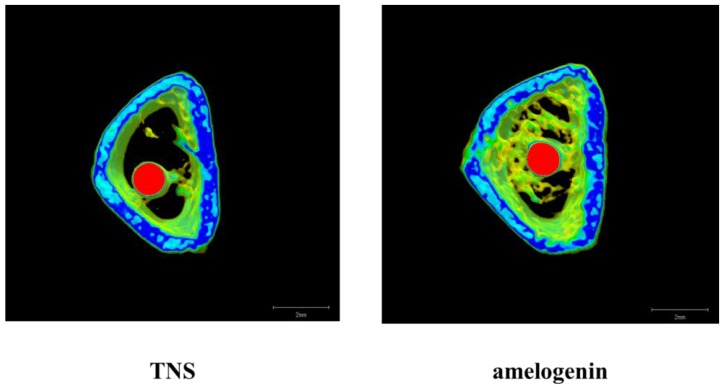
Micro-CT images (The implants were marked with red color, cortical bone with blue color, and cancellous bone with green color).

**Figure 13:**
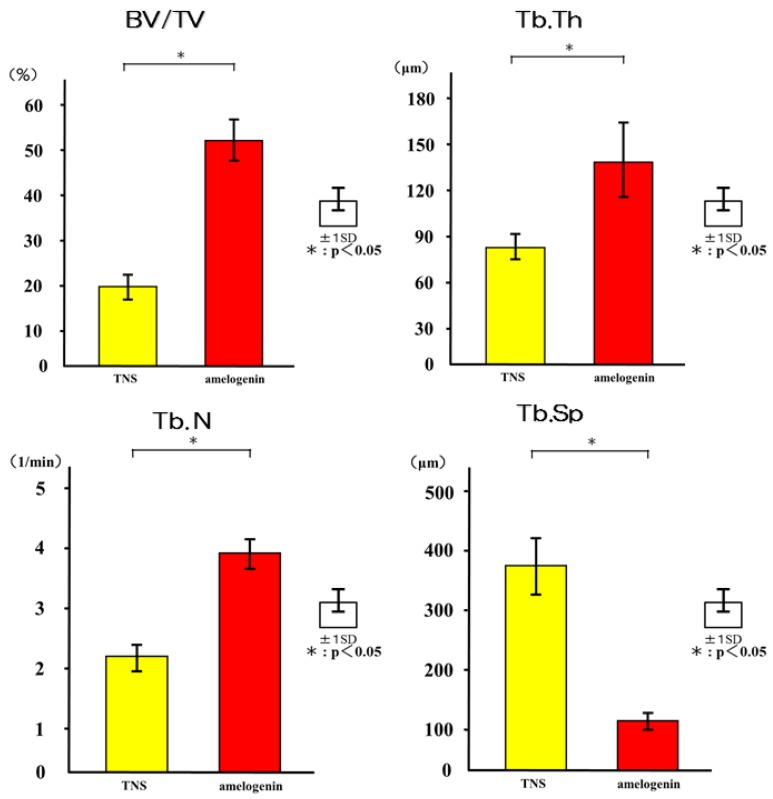
Quantitative evaluation of the trabecular bone within ROI (BV/TV, Tb.N, Tb.Th and Tb. Sp). * *p* < 0.05.

The reconstructed three-dimensional micro-computed tomography (CT) images from transverse slices of rat femurs including implants are shown in Figure 12. Both the test and control surface promoted new bone formation around the implants. More trabecular microarchitecture was observed in the vicinity of the amelogenin-coated TNS-modified titanium surface than of the TNS modified titanium surface. Quantitative evaluation of the trabecular bone within the region of interest (ROI) is shown in Figure 13. Bone volume (BV)/ tissue volume (TV), Tb.N, and Tb.Th were significantly higher in the test implant group compared to the control implant group (*p* < 0.05). Conversely, Tb.Sp exhibited a significantly lower value in the test group compared with the control group. 

**Figure 14:**
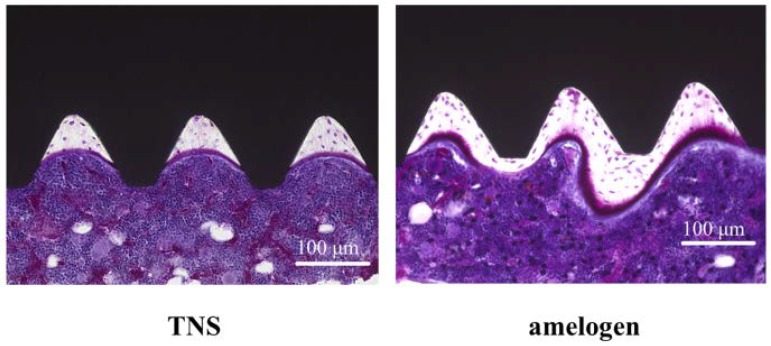
The longitudinally undecalcified histological sections with implants and peri-implant bones. (Purple color: osteoid, white color: new bone).

**Figure 15:**
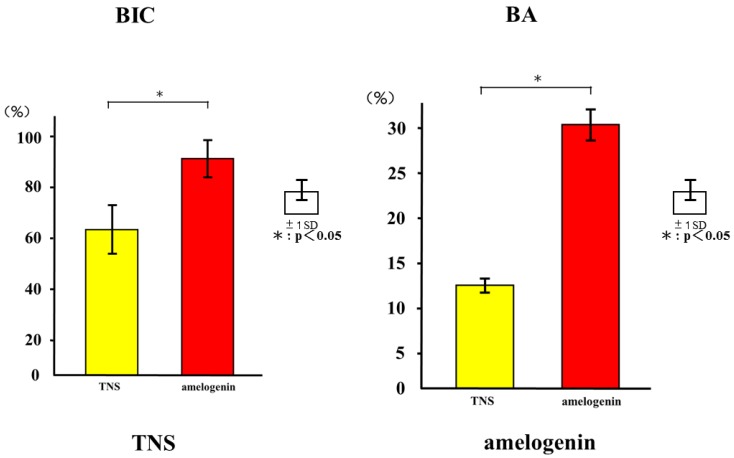
Quantitative histomorphometric analysis within the region of measurement above (BIC and BA). * *p* < 0.05.

The longitudinally un-decalcified histological sections with implants and peri-implant bones are presented in Figure 14. Apposition of new bone along the amelogenin-coated TNS-modified titanium implant surfaces was greater than that along the TNS-modified titanium implant surface, where only a small amount of new bone formed. Quantitative histomorphometric analysis within the region of measurement above (Figure 15) showed that the new bone area was significantly greater around the test implants than the control implants (*p* < 0.05). 

**Figure 16:**
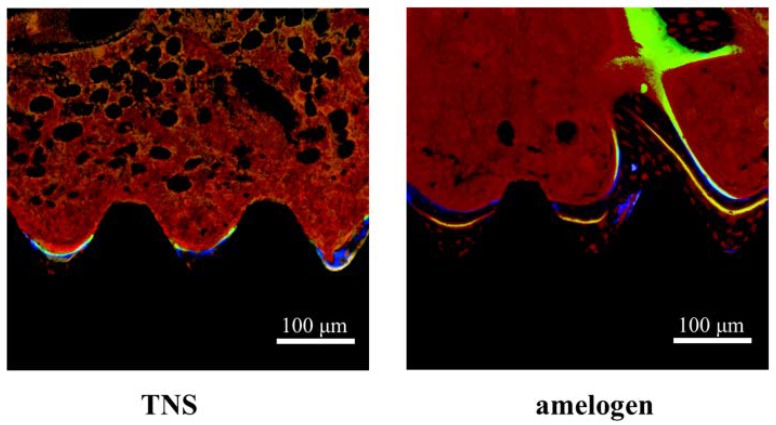
The histological sections were also observed using confocal laser scanning microscopy for dynamic histomorphometry according to fluorescence labeling. (Blue color: 1 week of new bone, yellow color: 4 week of new bone, green color: 8 week of new bone, red color: osteoid).

**Figure 17:**
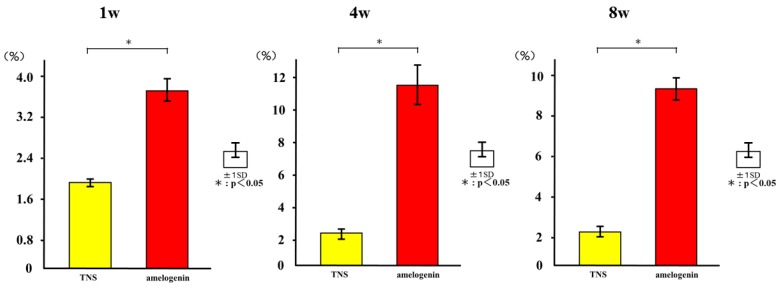
The percentage of labeled bone area (%LBA). * *p* < 0.05.

The histological sections were also observed using confocal laser scanning microscopy for dynamic histomorphometry according to fluorescence labeling (Figure 16). We observed colored linear signals, representing oxytetracycline hydrochloride (1 week), alizarin red S (4 weeks), and calcein (8 weeks) in new bone around the implants. The increases in the distance between the implant surface and labeled bone at different time points were significantly higher in the test implants than in the control implants. Quantitatively, the amelogenin-coated TNS-modified titanium implants exhibited the best effects with regard to increasing the percentage of labeled bone area (%LBA) compared with the control groups (*p* < 0.05) (Figure 17).

## 3. Discussion

In clinical dentistry, amelogenin, in the form of a commercial preparation of Emodogain^®^ (BIORA, Malmo, Sweden), is frequently used in the surgical process to induce RBM cell differentiation for cementgenesis and periodontal regeneration in patients with periodontal disease [27,28,29]. Therefore, based upon our previous findings that a titanium surface TNS-modified by alkali treatment could promote the initial attachment of RBM, RPL, and aortic endothelial cells, as well as bone differentiation both in vitro and in vivo, in the present study we investigated whether RBM and RPL cells might preferentially respond to titanium implants with an amelogenin-coated compared to an uncoated TNS-modified surface. By surface analysis, we confirmed that amelogenin could coat the TNS-modified titanium surface as a result of the TNS mechanical and chemical structure. We found that initial cell adhesion as well as the expression of RBM and RPL cell differentiation markers, such as ALP, Ca, and OCN, were elevated on alkali-treated titanium disks modified by amelogenin coating compared with TNS-modified titanium disks alone. Additionally, the results of PCR analysis suggested that the calcification derived from RPL cells induced by the amelogenin-coated TNS-modified titanium surface may be cementitious. The present study also provided the first evidence of enhanced bone volume in amelogenin-coated TNS-modified titanium implants compared with TNS-modified implants in vivo. The increased amount of new bone formation around the amelogenin-coated TNS-modified titanium may have occurred because of the amelogenin bound to the nanostructured surface, which activated osteogenic cells and accelerated subsequent bone mineralization. Our results therefore suggested that titanium disks modified by alkali treatment and amelogenin coating could promote RBM and RPL cell adhesion, differentiation, and activation, thereby augmenting calcium deposition both in vitro and in vivo.

The surface characteristics of titanium implants have been recognized as critical factors for achieving clinical success [7,8,9,10]. The nanotopogrphy on titanium surfaces plays important roles in modulating cell responses at the implant-tissue interface, which can have a large effect on tissue integration with the implant [1]. In the present study, we prepared TNS-modified titanium disks using the method established in our recent study [18], as we found that a TNS-modified titanium surface could promote the regulation of osteogenic differentiation of RBMs and enhance mineralization. Our results demonstrated that the TNS-modified titanium alloy disks were more hydrophilic and exhibited markedly improved wettability compared with unmodified surfaces. A better understanding of the surface roughness and topography of modified titanium alloy surfaces is needed to assess their wettability [32,33,34]. The nano-network structure formed on the titanium in the present study is similar to the hierarchical structure reported by Zhao et al. [35]. In their work, hierarchical nanotextured titanium alloy surface topographies with titania nanostructures that mimicked the hierarchical structure of bone tissues were produced by etching followed by anodization. Our research further revealed that NaOH treatment led to the formation of a Ti-O-Na titanate layer on the titanium surface, likely by causing a thick oxide film to form on the titanium surface TiO_2_ layer. 

Moreover, Tashiro et al. previously fabricated a TNS-modified titanium QCM sensor [36]. A QCM sensor is a profoundly delicate and convenient device that is used to observe protein adsorption and cell behavior in situ, which is comprised of a quartz crystal and a detection material. A 27-MHz QCM can provide highly sensitive measurements of mass in aqueous solutions, as the resonance frequency decreases in relation to the mass of the protein bound on the QCM electrode surface. These previous findings support the potential utility of the QCM method for the evaluation of protein adsorption behaviors on implant surfaces. In the present study, the amount of amelogenin adsorption on the TNS-modified titanium was higher than that of the unmodified titanium sensor. Notably, the wettability of a material surface constitutes an important factor for the adsorption of proteins as determined by the protein adsorption principle [34,37,38]. Protein adsorption rate on the TNS was correlated with the contact angle, suggesting that the hydrophilicity of titanium greatly affected its protein adsorption ability. The contact angle of the TNS and Ti sensors fluctuated in the hydrophilic range, with hydrophilicity of the surfaces expanding after treatment, corresponding to the formation of the TNSs [18]. Thus, the growth of the titanium oxide layer increased the surface energy, resulting in a more hydrophilic surface [18,36]. In addition, the results of SEM, SPM, XPS, and FTIR analyses confirmed that the nano-network structure of the titanium surface was modified by an amelogenin thin film owing to the potential of TNS. Amelogenin has been shown by Hoang et al. to exhibit several characteristics of the surface adherent material class of cell adhesion proteins [24,39]. However, as amelogenin does not contain an RGD or various other specific sequences, the cell-surface receptor for amelogenin may not be an integrin [7]. Furthermore, unlike fibronectin, which contains an RGD sequence, amelogenin can bind to cells or material surface without heparin. Therefore, the amelogenin coated on TNS-modified titanium surfaces through nanostructures may be useful as self-scaffolds.

The amelogenin-coated TNS-modified titanium surface increased ALP activity, OCN production, calcium deposition, and osteogenesis-related gene expression in RBM cells. ALP activity is a biological marker of bone differentiation at early stages of differentiation as well as of bone formation and osteoblast activity [40]. Osteoblasts produce and incorporate OCN into the bone matrix; OCN is released into the circulation from the matrix during bone resorption and as such, is considered as a marker of bone turnover rather than of bone formation [41]. We observed differences in the expression levels of osteoblast-specific markers between amelogenin-coated and uncoated TNS-modified titanium implant surfaces. In addition, the observed upregulation in Runx2 and BMP, which are key transcription factors mediating osteoblast differentiation, as well as ALP and OPN levels in RBM cells grown on the alkali-treated surface reflected its greater capacity for inducing osteogenic differentiation [42,43,44]. This is consistent with findings of Kawana et al. and Shimizu et al. [45,46], who reported the ability of amelogenin in EMD to enhance bone regeneration in rat femur and around titanium implants in rats. Analysis of our data suggested that amelogenin maintains the viability of adherent stromal cells and promotes their osteoblastic differentiation.

Some studies show that enamel matrix proteins such as amelogenin induce cementum formation and achieve periodontium repair in rats, monkeys, and humans [47,48,49]. Amelogenin stimulates RPL cells to induce periodontium regeneration and the formation of new cementum, bone, and dentin in clinical trials. In the present study, amelogenin coating on a TNS-modified titanium surface promoted the upregulation of RPL cell attachment and proliferation, as well as bone differentiation. In particular, CEMP-1 may play a role as a local regulator of cementoblast differentiation and cementum-matrix mineralization. This protein was shown to be expressed by cementoblasts and progenitor cells localized in the RPL cells. To confirm the formation of cementum, which constitutes one of the periodontal tissues, ALP activity, Runx2, and BMP served as useful key factors at the in vitro level. Our experimental results suggested that amelogenin induced not only new bone but also the formation of cementum.

The rat femur model in the present study was employed to evaluate the bone tissue response to implants via only trabecular bone, which is more relevant to clinical situations. The bone formation activity around the amelogenin-coated TNS-modified titanium implant was continuous and substantial up to week 8 in the healing process. In contrast, bone formation activity barely increased around the TNS-modified implant without amelogenin. Healing at this time point is considered to be in the final stage of wound healing in rat models [50,51], whereas the degree of bone–implant contact is affected by the early bone response to implant surface. In the present study, the bone-implant contact pattern for the amelogenin-coated TNS-modified titanium was enhanced compared with that of the TNS-modified implants alone. Emdogain^®^-provided amelogenin may exert a survival and a differentiative signal on RBM cells, an effect which fits well with its ascribed role in promoting early stages of in vivo bone formation as reported by Kawana et al. [45]. This study supports our present research, confirming that an amelogenin-coated TNS-modified titanium surface promotes bone differentiation around titanium implants not only at the in vitro level but also in vivo. Further studies are necessary to analyze the mechanism of induction utilized by the amelogenin-coated TNS-modified titanium surface.

## 4. Materials and Methods 

### 4.1. Sample Preparation

Titanium samples (JIS Grade 2, 15 mm in diameter and 1 mm-thick titanium, Daido Steel, Osaka, Japan) and titanium screw implants (1.2 mm in external diameter and 12 mm in length, Daido Steel, Osaka, Japan) treated with NaOH to form TNSs on their surfaces, were used as the experimental material. Samples were then dried at room temperature. Disks of 316 were divided into the test and control groups. The disks were immersed in 10 M aq. NaOH and maintained at 30 °C for 24 h. The solution in each flask was replaced with distilled water (200 mL) until the solution reached a conductivity of 5 μS/cm. Samples were then dried at room temperature. TNS-modified titanium disks and titanium screws were incubated with high concentration (4 μg/mL) amelogenin (Amelogenin, X isoform isoform 1, ATGen Co., Ltd., Filgen, Aichi, Japan) at 37 °C for 3 h. Amelogenin was further aggregated on the surface of titanium disks and screws through lyophilization (Freeze-Dry). Because amelogenin molecules aggregate through their C-terminal hydrophobic domain, the attached amelogenin is released gradually. Amelogenin coated on TNS-modified titanium samples comprised the test group, and TNS-modified titanium samples alone served as the control group.

### 4.2. Characterization of Materials

The surface topography of the samples was qualitatively evaluated by scanning electron microscopy (SEM, S-4000, Shimadzu, Kyoto, Japan) and scanning probe microscopy (SPM; SPM-9600, Shimadzu). The composition of the coating was analyzed by X-ray photoelectron spectroscopy (XPS; Kratos Analytical Axis ultra DLD electron spectrometer, Kratos Instruments, Manchester, UK) using a monochromatic Al Kα X-ray source. Amelogenin-coated TNS disks were characterized by Fourier transform-infrared (FTIR) spectroscopy (Spectrum One, Perkin Elmer Co. Norwalk, CT, USA). 

### 4.3. QCM Measurements

To evaluate the effects of nano-modified titanium surfaces on the adsorption of amelogenin, we used a QCM sensor. A Ti QCM sensor was fabricated by reactive magnetron sputtering. Specifically, a thin layer of Ti was deposited on the QCM sensor. This sensor was then alkali-modified by treatment with NaOH at room temperature to fabricate TNS. The amounts of protein (amelogenin) were determined by QCM measurements (Affinix QN μ; Initium Co., Ltd., Tokyo, Japan) The Affinix QN μ utilized a 550 µL cell outfitted with a 27 MHz QCM plate at the base of the cell. The diameter of the quartz plate was 8 mm, and the area of the gold-plated quartz was 4.9 mm^2^. The unit also included a mixing bar and a temperature controller. The adjustment in recurrence was checked utilizing a universal frequency counter connected to a microcomputer. 

The Ti QCM sensors and TNS were immersed in 500 μL of phosphate buffered saline (PBS; 0.01 M PBS at pH 7.4). Changes in the QCM frequency were measured as a function of time; recording started immediately after the infusion of 5 μL (20 μg/mL) amelogenin. The solution was mixed to avoid any influence of protein dispersion on the measured results. Stirring did not influence the soundness of the frequency or the degree of frequency adjustment. The frequency change relied upon the adsorbed mass in accordance with the Sauerbrey equation.

### 4.4. Cell Culture

RBM cells were isolated from the femurs of 7-week-old Sprague-Dawley rats. Briefly, rats were euthanized using 4% isoflurane, and the bones were aseptically excised from the hind limbs. The proximal end of the femur and the distal end of the tibia were clipped. A 21-gauge needle (Terumo, Tokyo, Japan) was inserted into the hole in the knee joint of each bone and the marrow was flushed from the shaft with culture medium. The resulting marrow pellet was dispersed by trituration and the cell suspensions from all bones were combined in a centrifuge tube. RBM cells were cultured in 75 cm^2^ culture flasks (Falcon, Becton Dickinson Labware, Franklin Lakes, NJ, USA) in culture medium.

RPL cells were purchased from Lonza (Walkersville, MD, USA). The medium (BulletKit™; Lonza, Basal, Switzerland) was prepared at an appropriate concentration (1 mL/cm^2^) and the cells were carefully thawed from cryovials. According to the recommended seeding density (3500 cells/cm^2^), the cells were placed into prepared tissue culture flasks and incubated in a 5% CO_2_ humidified incubator at 37 °C. The growth medium was changed the day after seeding and every other day thereafter. When the cells reached 70–80% confluency throughout the flask, they were subcultured using 4-(2-hydroxyethyl)-1-piperazineethanesulfonic acid-buffered saline solution, trypsin/ethylenediaminetetraacetic acid, and trypsin neutralizing solution. Then, the harvested cells were pelleted by centrifugation at 220× *g* for 5 min. 

At confluence, cells were removed from flasks by trypsinization, washed twice with PBS (Dulbecco’s Formula Modified, ICN Biochemicals, Berks, UK), resuspended in culture medium, and seeded at a cell density of 4 × 10^4^ cells/cm^2^ into 24-well tissue culture plates (Falcon) containing test or control titanium disks. The cells were cultured at 37 °C in a humidified 5% CO_2_/95% air atmosphere. This study was performed according to the Guidelines for Animal Experimentation at Osaka Dental University (Approval No. 16-05001, 1 May 2016).

### 4.5. Cell Adhesion

RBM and RPL cell adhesion was evaluated using the CellTiter-Blue Cell Viability Assay kit (Promega, Madison, WI, USA) according to the manufacturer’s protocol. Briefly, RBM cells were seeded on samples at a density of 4 × 10^4^/cm^2^ and allowed to attach for 1, 3, 6, and 24 h. After 24 h, test and control samples were stained with Alexa Fluor 488-phalloidin (Invitrogen/Life Technologies, Carlsbad, CA, USA) and DAPI. F-actin and cell nuclei were visualized by confocal laser scanning microscopy (LSM700; Carl Zeiss, Oberkochen, Germany).

### 4.6. Amelogenin-Induced RBM and RPL Cell Differentiation and Matrix Deposition on TNS-Modified Titanium Surfaces In Vitro 

Total RNA was extracted from cells and 1 μg was used to synthesize cDNA with a High-Capacity cDNA Archive kit (Applied Biosystems, Foster City, CA, USA) after 3, 7, 14, and 21 days. Alkaline phosphatase (Alp), runt-related transcription factor (Runx2), bone morphogenetic protein (Bmp), and osteopontin (Opn) mRNA (RBM), or Alp, Opn, Bmp, and cementum protein 1 (CEMP-1) mRNA (RPL) expression was investigated by quantitative reverse transcription-polymerase chain reaction (qRT-PCR) using a StepOne Plus Real-Time RT-PCR system (Applied Biosystems). 

RBM cells were washed with PBS and lysed with 200 μL of 0.2% Triton X-100 (Sigma-Aldrich, St. Louis, MO, USA). ALP activity was measured using the Alkaline Phosphatase Luminometric enzyme-linked immunosorbent assay (ELISA) kit (Sigma-Aldrich) according to the manufacturer’s protocol after 7 and 14 days of culture. DNA content was measured using the PicoGreen dsDNA Assay kit (Invitrogen/Life Technologies) according to the manufacturer’s protocol. The amount of ALP was normalized to the amount of DNA in the cell lysate.

After 21 and 28 days of culture, sandwich ELISA was used to determine OCN levels directly in the cell culture supernatant via a commercial kit (Rat Osteocalcin ELISA kit; DS Pharma Biomedical Co., Osaka, Japan) according to the manufacturer’s instructions.

Mineralization was assessed using a Calcium E-test kit (Wako Pure Chemical Industries, Otsu, Shiga, Japan) at each point (21 or 28 days of culture). The concentration of calcium ions was calculated according to the manufacturer’s instructions.

### 4.7. Animal Model and Surgical Procedures

A total of 20 male SD rats (Shimizu Laboratory Supplies Co., Kyoto, Japan; age 8 weeks, weighing 160 ± 15 g) were used in this study. The experimental animals were randomly divided into two groups, with 10 rats in each group. The experimental method is based on the previous experience of our research group [11,32]. Animals were administered inhalation anesthesia, followed by intraperitoneal injection of anesthetics (1.5 mL/kg). The hair was shaved off the right hind limb and the skin was disinfected with iodine followed by 75% ethanol to remove the iodine. A 1 cm-long longitudinal skin incision was made along the medial side of the knee joint and the subcutaneous fascia was incised. The patella and extensor mechanism were then dislocated to expose the distal aspect of the femur. A pilot hole was drilled through the intercondylar notch using a 1 mm-round dental burr under profuse sterile saline irrigation, and the hole was enlarged to 1.2 mm with an endodontic file. The implants, sterilized by ethylene oxide gas, were randomly inserted into the 20 prepared channels and the medullary cavities of the right femurs. After surgery, the knee joint was restored and the surgical site was closed in layers. The animals received intramuscular injections of gentamicin (1 mg/kg) and buprenorphine (0.05 mg/kg) for 3 days to prevent postsurgical infection and relieve pain. All rats were allowed free movement without any restriction. 

### 4.8. Sequential Fluorescence Labeling

Polychrome sequential labeling of bone using intraperitoneal injection of fluorochromes was performed to record the process of new bone formation and mineralization after the implantation according to the following schedule: first week, oxytetracycline hydrochloride at 25 mg/kg (Sigma-Aldrich); fourth week, alizarin red S at 30 mg/kg (Sigma-Aldrich); and eighth week, calcein at 20 mg/kg (Sigma-Aldrich). All animals were sacrificed by an intraperitoneally injected overdose of sodium pentobarbital 3 days after the final labeling treatment.

### 4.9. Amelogenin-Induced Bone Differentiation on the TNS-Modified Titanium Surface In Vivo 

Immediately after dissection, the right femurs including the implants were placed in cool saline solution and scanned with an SMX-130CT micro-computed tomography (micro-CT) scanner (Shimadzu) operated at 70 kV and 118 mA; the isotropic voxel size was 10 μm in all spatial directions. After tomographic acquisitions, the implant and surrounding tissue were reconstructed and analyzed using morphometric software (TRI/3D-BON; Ratoc System Engineering, Tokyo, Japan). The region of interest (ROI) was defined as the 500 μm-wide area of bone around the implants from 2 mm below the highest point of the growth plate to the distal 100 slices [32]. The bone volume fraction (BV/TV), mean trabecular number (Tb.N), mean trabecular thickness (Tb.Th), and mean trabecular separation (Tb.Sp) were calculated within the ROI.

After the micro-CT scan, the femoral specimens were used to create undecalcified histological sections. The specimens were fixed in 70% ethanol solution for 7 days followed by immersion in Villanueva bone stain solution [33]. The sections were histomorphometrically analyzed using a BZ-9000 digital microscope (Keyence Co., Osaka, Japan). Fluorescence microscopic evaluation was also performed using a confocal laser scanning microscope (LSM 700). The excitation/emission wavelengths of the chelating fluorochromes were 351/460 nm, 543/617 nm, and 488/517 nm for oxytetracycline hydrochloride (blue), alizarin red S (red), and calcein (green), respectively. The region of measurement was defined on sections approximately 2 mm below the growth plate to 1 mm distally according to the micro-CT analysis.

### 4.10. Statistical Analyses

All samples were prepared four times. Data are presented as the means ± standard deviation. In all analyses, statistical significance was determined using the paired two-tailed Student’s *t*-test. A P value <0.05 was considered statistically significant.

## 5. Conclusions

Our results showed that an amelogenin-coated TNS-modified titanium surface promoted attachment and bone differentiation of RBM and RPL cells along with periodontal regeneration in vitro, and induced bone differentiation around titanium implants in vivo. Combination of amelogenin and nano-structure on a titanium surface, thus, constitutes a promising approach to facilitate osseoinetgration around titanium implants and bone tissue engineering.

## Abbreviations

ALP	alkaline phosphatase
BMP	bone morphogenetic protein
DAPI	4′,6-diamidino-2-phenylindole
EMD	enamel matrix derivative
FTIR	Fourier transform-infrare
OCN	osteocalcin
OPN	osteopontin
PBS	phosphate-buffered saline
QCM	Quartz crystal microbalance
RBM	rat bone marrow
RPL	rat periodontal ligament
Runx2	runt-related transcription factor 2
SEM	scanning electron microscopy
SPM	scanning probe microscopy
TiO_2_	titania
TNS	titania nano sheet
XPS	X-ray photoelectron spectroscopy

## References

[1] Moradian-Oldak J., Wen H.B., Schneider G.B., Stanford C.M. (2006). Tissue engineering strategies for the future generation of dental implants. Periodontology 2000.

[2] Kirsch A. (1983). The two-phase implantation method using IMZ intramobile cylinder implants. J. Oral Implantol..

[3] Skalak R. (1983). Biomechanical considerations in osseointegrated prostheses. J. Prosthet. Dent..

[4] Buser D., Warrer K., Karring T. (1990). Formation of a periodontal ligament around titanium implants. J. Periodontol..

[5] Choi B.H. (2000). Periodontal ligament formation around titanium implants using cultured periodontal ligament cells: A pilot study. Int. J. Oral Maxillofac. Implants.

[6] Hasegawa M., Yamato M., Kikuchi A., Okano T., Ishikawa I. (2005). Human periodontal ligament cell sheets can regenerate periodontal ligament tissue in an athymic rat model. Tissue Eng..

[7] Boyan B.D., Hummert T.W., Dean D.D., Schwartz Z. (1996). Role of material surfaces in regulating bone and cartilage cell response. Biomaterials.

[8] Schwartz Z., Lohmann C.H., Oefinger J., Bonewald L.F., Dean D.D., Boyan B.D. (1999). Implant surface characteristics modulate differentiation behavior of cells in the osteoblastic lineage. Adv. Dent. Res..

[9] Takeuchi K., Saruwatari L., Nakamura H.K., Yang J.M., Ogawa T. (2005). Enhanced intrinsic biomechanical properties of osteoblastic mineralized tissue on roughened titanium surface. J. Biomed. Mater. Res. A.

[10] Ogawa T., Ozawa S., Shih J.H., Ryu K.H., Sukotjo C., Yang J.M., Nishimura I. (2000). Biomechanical evaluation of osseous implants having different surface topographies in rats. J. Dent. Res..

[11] Ogawa T., Nishimura I. (2003). Different bone integration profiles of turned and acid-etched implants associated with modulated expression of extracellular matrix genes. Int. J. Oral Maxillofac. Implants.

[12] Liu X., Chu P.K., Ding C. (2004). Surface modification of titanium, titanium alloys, and related materials for biomedical applications. Mater. Sci. Eng. R Rep..

[13] Martin J.Y., Schwartz Z., Hummert T.W., Schraub D.M., Simpson J., Lankford J., Dean D.D., Cochran D.L., Boyan B.D. (1995). Effect of titanium surface roughness on proliferation, differentiation, and protein synthesis of human osteoblast-like cells (MG63). J. Biomed. Mater. Res..

[14] Hamlet S., Alfarsi M., George R., Ivanovski S. (2012). The effect of hydrophilic titanium surface modification on macrophage inflammatory cytokine gene expression. Clin. Oral Implants Res..

[15] Cooper L.F., Masuda T., Yliheikkilä P.K., Felton D.A. (1997). Generalizations regarding the process and phenomenon of osseointegration. Part II. In vitro studies. Int. J. Oral Maxillofac. Implants.

[16] Setzer B., Bächle M., Metzger M.C., Kohal R.J. (2009). The gene-expression and phenotypic response of hFOB 1.19 osteoblasts to surface-modified titanium and zirconia. Biomaterials.

[17] Kasuga T., Hiramatsu M., Hoson A., Sekino T., Niihara K. (1999). Titania nanotubes prepared by chemical processing. Adv. Mater..

[18] Komasa S., Taguchi Y., Nishida H., Tanaka M., Kawazoe T. (2012). Bioactivity of nanostructure on titanium surface modified by chemical processing at room temperature. J. Prosthodont. Res..

[19] Fujino T., Taguchi Y., Komasa S., Sekino T., Tanaka M. (2014). Cell differentiation on nanoscale features of a titanium surface: Effects of deposition time in NaOH solution. J. Hard Tissue Biol..

[20] Xing H., Komasa S., Taguchi Y., Sekino T., Okazaki J. (2014). Osteogenic activity of titanium surfaces with nanonetwork structures. Int. J. Nanomed..

[21] Zhang H., Komasa S., Mashimo C., Sekino T., Okazaki J. (2017). Effect of ultraviolet treatment on bacterial attachment and osteogenic activity to alkali-treated titanium with nanonetwork structures. Int. J. Nanomed..

[22] Nakano Y., Komasa S., Taguchi Y., Sekino T., Okazaki J. (2013). Rat endothelial cell attachment, behavior and gene expression on NaOH-treated titanium surfaces. J. Oral Tissue Eng..

[23] Hara Y., Komasa S., Yoshimine S., Nishizaki H., Okazaki J. (2018). Effect of nano modified titanium surface on adsorption of rat periodontal ligament cells. J. Osaka Dent. Univ..

[24] Hoang A.M., Klebe R.J., Steffensen B., Ryu O.H., Simmer J.P., Cochran D.L. (2002). Amelogenin is a cell adhesion protein. J. Dent. Res..

[25] Fukumoto S., Kiba T., Hall B., Iehara N., Nakamura T., Longenecker G., Krebsbach P.H., Nanci A., Kulkarni A.B., Yamada Y. (2004). Ameloblastin is a cell adhesion molecule required for maintaining the differentiation state of ameloblasts. J. Cell Biol..

[26] Gibson C.W. (2008). The amelogenin “enamel proteins” and cells in the periodontium. Crit. Rev. Eukaryot. Gene Expr..

[27] Zetterström O., Andersson C., Eriksson L., Fredriksson A., Friskopp J., Heden G., Jansson B., Lundgren T., Nilveus R., Olsson A. (1997). Clinical safety of enamel matrix derivative (EMDOGAIN) in the treatment of periodontal defects. J. Clin. Periodontol..

[28] Sculean A., Chiantella G.C., Windisch P., Donos N. (2000). Clinical and histologic evaluation of human lntrabony defects treated with an enamel matrix protein derivative (Emdogain). Int. J. Periodontics Restor. Dent..

[29] Esposito M., Grusovin M.G., Coulthard P., Worthington H.V. (2005). Enamel matrix derivative (Emdogain) for periodontal tissue regeneration in intrabony defects. Cochrane Database Syst. Rev..

[30] Gestrelius S., Lyngstadaas S.P., Hammarström L. (2000). Emdogain–periodontal regeneration based on biomimicry. Clin. Oral Investig..

[31] Bosshardt D.D. (2008). Biological mediators and periodontal regeneration: A review of enamel matrix proteins at the cellular and molecular levels. J. Clin. Periodontol..

[32] Kusumoto T., Yin D., Zhang H., Chen L., Nishizaki H., Komasa Y., Okazaki J., Komasa S. (2017). Evaluation of the osteointegration of a novel alkali-treated implant system in vivo. J. Hard Tissue Biol..

[33] Xu L.C., Siedlecki C.A. (2007). Effects of surface wettability and contact time on protein adhesion to biomaterial surfaces. Biomaterials.

[34] Zhao L., Chu P.K., Zhang Y., Wu Z. (2009). Antibacterial coatings on titanium implants. J. Biomed. Mater. Res. B Appl. Biomater..

[35] Tashiro Y., Komasa S., Miyake A., Nishizaki H., Okazaki J. (2018). Analysis of titania nanosheet adsorption behavior using a quartz crystal microbalance sensor. Adv. Mater. Sci. Eng..

[36] Dowling D.P., Miller I.S., Ardhaoui M., Gallagher W.M. (2011). Effect of surface wettability and topography on the adhesion of osteosarcoma cells on plasma-modified polystyrene. J. Biomater. Appl..

[37] Wei J., Igarashi T., Okumori N., Igarashi T., Maetani T., Liu B., Yoshinari M. (2009). Influence of surface wettability on competitive protein adsorption and initial attachment of osteoblasts. Biomed. Mater..

[38] Du C., Schneider G.B., Zaharias R., Abbott C., Seabold D., Stanford C., Moradian-Oldak J. (2005). Apatite/amelogenin coating on titanium promotes osteogenic gene expression. J. Dent. Res..

[39] Kodama H.A., Amagai Y., Sudo H., Kasai S., Yamamoto S. (1981). Establishment of a clonal osteogenic cell line from newborn mouse calvaria. Jpn. J. Oral Biol..

[40] Ayukawa Y., Takeshita F., Inoue T., Yoshinari M., Shimono M., Suetsugu T., Tanaka T. (1998). An immunoelectron microscopic localization of noncollagenous bone proteins (osteocalcin and osteopontin) at the bone–titanium interface of rat tibiae. J. Biomed. Mater. Res..

[41] Guo J., Padilla R.J., Ambrose W., De Kok I.J., Cooper L.F. (2007). The effect of hydrofluoric acid treatment of TiO2 grit blasted titanium implants on adherent osteoblast gene expression in vitro and in vivo. Biomaterials.

[42] Isa Z.M., Schneider G.B., Zaharias R., Seabold D., Stanford C.M. (2006). Effects of fluoride-modified titanium surfaces on osteoblast proliferation and gene expression. Int. J. Oral Maxillofac. Implants.

[43] Marinucci L., Balloni S., Becchetti E., Belcastro S., Guerra M., Calvitti M., Lull C., Calvi E.M., Locci P. (2006). Effect of titanium surface roughness on human osteoblast proliferation and gene expression in vitro. Int. J. Oral Maxillofac. Implants.

[44] Kawana F., Sawae Y., Sahara T., Tanaka S., Debari K., Shimizu M., Sasaki T. (2001). Porcine enamel matrix derivative enhances trabecular bone regeneration during wound healing of injured rat femur. Anat. Rec..

[45] Shimizu-Ishiura M., Tanaka S., Lee W.S., Debari K., Sasaki T. (2002). Effects of enamel matrix derivative to titanium implantation in rat femurs. J. Biomed. Mater. Res..

[46] Slavkin H.C., Bessem C., Fincham A.G., Bringas P., Santos V., Snead M.L., Zeichner-David M. (1989). Human and mouse cementum proteins immunologically related to enamel proteins. Biochim. Biophys. Acta.

[47] Viswanathan H.L., Berry J.E., Foster B.L., Gibson C.W., Li Y., Kulkarni W.B., Snead M.L., Somerman M.J. (2003). Amelogenin: A potential regulator of cementum-associated genes. J. Periodontol..

[48] Hammarstrom L. (2008). The role of enamel matrix proteins in the development of cementum and periodontal tissues. Ciba Foundation Symposium 205-Dental Enamel.

[49] Brunette D.M., Chehroudi B. (1999). The effects of the surface topography of micromachined titanium substrata on cell behavior in vitro and in vivo. J. Biomech. Eng..

[50] Sikavitsas V.I., van den Dolder J., Bancroft G.N., Jansen J.A., Mikos A.G. (2003). Influence of the in vitro culture period on the in vivo performance of cell/titanium bone tissue-engineered constructs using a rat cranial critical size defect model. J. Biomed. Mater. Res..

[51] Ferris D.M., Moodie G.D., Dimond P.M., Giorani C.W., Ehrlich M.G., Valentini R.F. (1999). RGD-coated titanium implants stimulate increased bone formation in vivo. Biomaterials.

